# Supplementary figures and images for: Utilization of CoRDS registry to monitor quality of life in patients with VCP multisystem proteinopathy
Source: Orphanet J Rare Dis. 2025 Apr 15;20:178. doi: 10.1186/s13023-025-03567-w (PMC11998231; doi:10.1186/s13023-025-03567-w)

## Slide 1
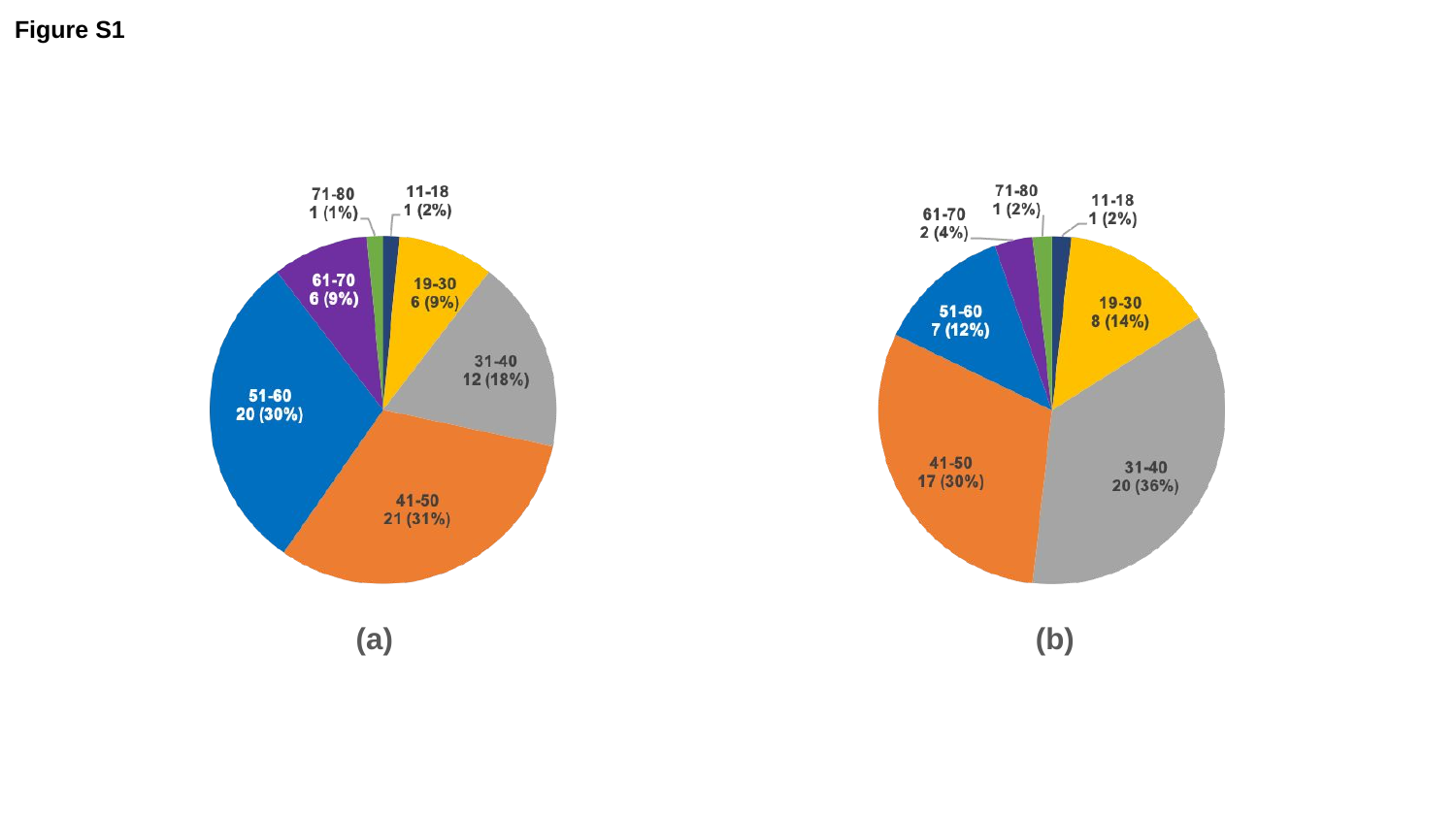

Figure S1
(a)
(b)

Supplement: Supplementary file 1 — Supplementary Material 1: Figure 1. Demographics. (a) Age of diagnosis of participants with VCP disease, (b) Age of first symptom onset in VCP disease participants. Each slice represents a specific age range. [file 13023_2025_3567_MOESM1_ESM.pptx]
