# Supplementary material for: Utilization of CoRDS registry to monitor quality of life in patients with VCP multisystem proteinopathy
Source: Orphanet J Rare Dis. 2025 Apr 15;20:178. doi: 10.1186/s13023-025-03567-w (PMC11998231; doi:10.1186/s13023-025-03567-w)
Supplement: Supplementary file 2 — Supplementary Material 2: Figure 2. Quality of Life - A comparison of pre-symptomatic vs. symptomatic VCP patients. A comparison between pre-symptomaticand symptomaticparticipants when asked five QOL questions was conducted: (a) Participants’ responses when asked about their general health, (b) Participants’ responses when asked if their health limits them in doing vigorous activities, (c) Participants’ responses when asked if they feel depressed. (d) Participants’ responses when asked how often they feel tired, (e) Participants’ responses when asked how much pain interfered with their enjoyment of life. [file 13023_2025_3567_MOESM2_ESM.pptx]

## Slide 1
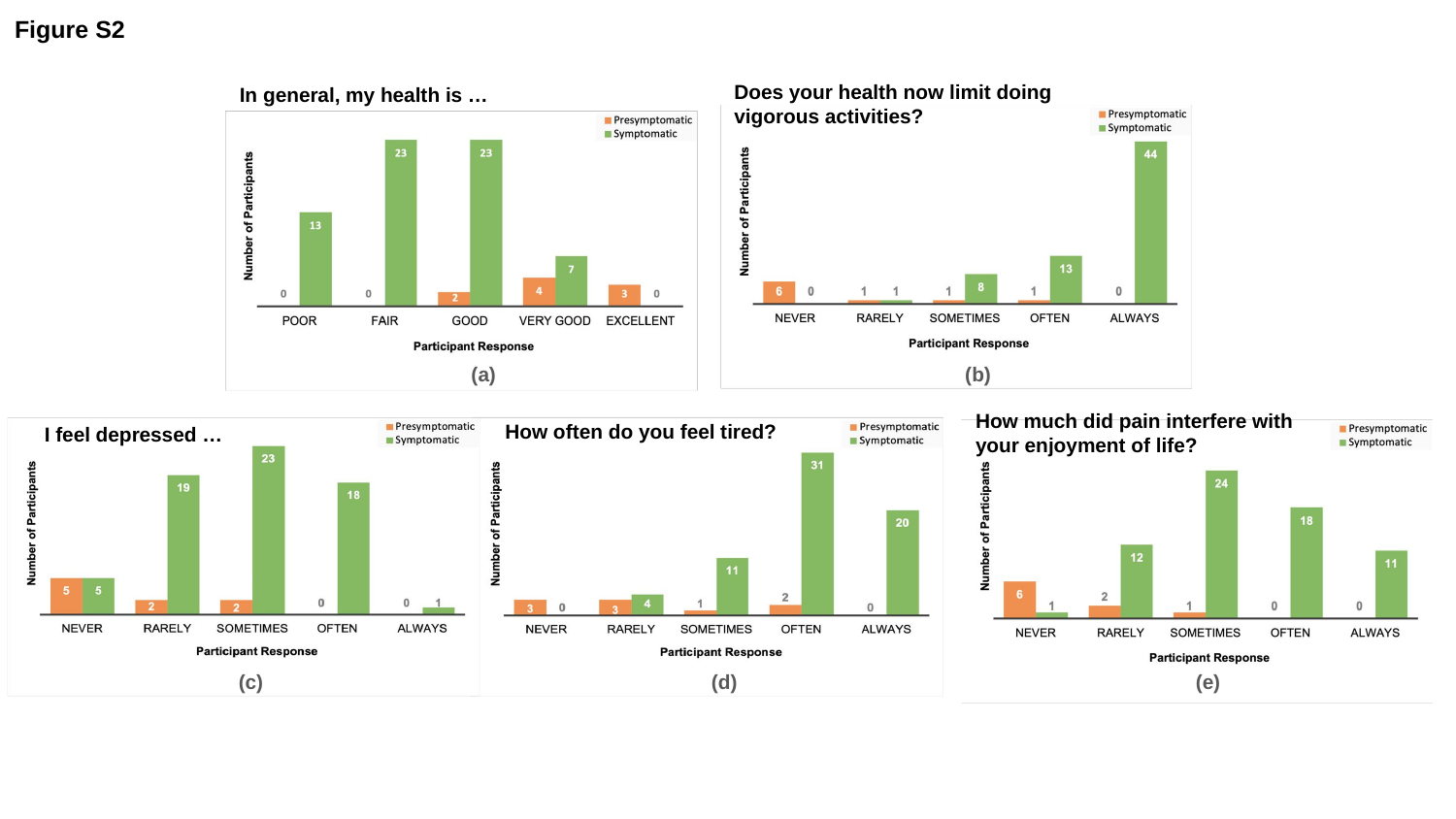

Figure S2
Does your health now limit doing
vigorous activities?
In general, my health is …
(a)
(b)
How much did pain interfere with
your enjoyment of life?
How often do you feel tired?
I feel depressed …
(c)
(d)
(e)
